# Acneiform drug eruptions—update on pathophysiology and culprit drugs

**DOI:** 10.3389/fmed.2026.1769362

**Published:** 2026-02-23

**Authors:** Paul Ulrich, Konstantin Drexler, Mark Berneburg, Bernadett Kurz, Dennis Niebel

**Affiliations:** 1 Department of Dermatology, University Hospital Regensburg, Regensburg, Germany; 2 Heinz-Werner-Seifert-Institute for Dermatopathology KölnBonn, Bonn, Germany

**Keywords:** acne, acneiform eruption, adverse drug reactions, rosacea, skin-directed therapy, targeted therapies

## Abstract

Acneiform adverse drug reactions (ADRs) appear within weeks to months after the initiation of a new medication. As opposed to acne vulgaris, they typically present monomorphic inflammatory lesions, may involve atypical, non-seborrheic body sites and may arise outside the usual age range of any acne subtype. A wide range of drugs can trigger the eruption of acneiform ADRs with targeted therapies used in oncology (e.g., epidermal growth factor receptor inhibitors) carrying the highest risk. More recently, an increasing incidence of acneiform ADRs has been observed with the advent of januskinase inhibitors now being used in a wide range of inflammatory conditions. Many times, symptomatic treatment rather than discontinuation of the causative drug is feasible, particularly when the medication is essential for managing a serious underlying condition. Close cooperation between dermatologists and prescribing specialists is essential to manage cutaneous side effects enabling maintenance of the best available therapeutic regimen.

## 1 Introduction

Within the broad spectrum of adverse drug reactions (ADRs), the skin and mucosa are affected very frequently. Cutaneous adverse drug reactions (cADRs) may be classified according to their underlying mechanism (immunologic vs. non-immunologic), their clinical presentation (e.g., maculopapular exanthema, symmetrical drug-related intertriginous and flexural exanthema, fixed drug eruption), or their histopathologic pattern (e.g., spongiotic, psoriasiform, lichenoid, or granulomatous drug eruptions). Non-immunologic cADRs are common and often reflect off-target pharmacologic effects, such as retinoid-induced xerosis, whereas immunologic cADRs are less frequent and correspond to the Coombs and Gell classification, with type IV reactions being most prevalent. Besides that, severe cutaneous adverse drug reactions (SCAR) comprise Stevens–Johnson syndrome (SJS), toxic epidermal necrolysis (TEN), drug reaction with eosinophilia and systemic symptoms/drug hypersensitivity syndrome (DRESS/DHS), and acute generalized exanthematous pustulosis (AGEP) (1, 2).

**Figure 1:**
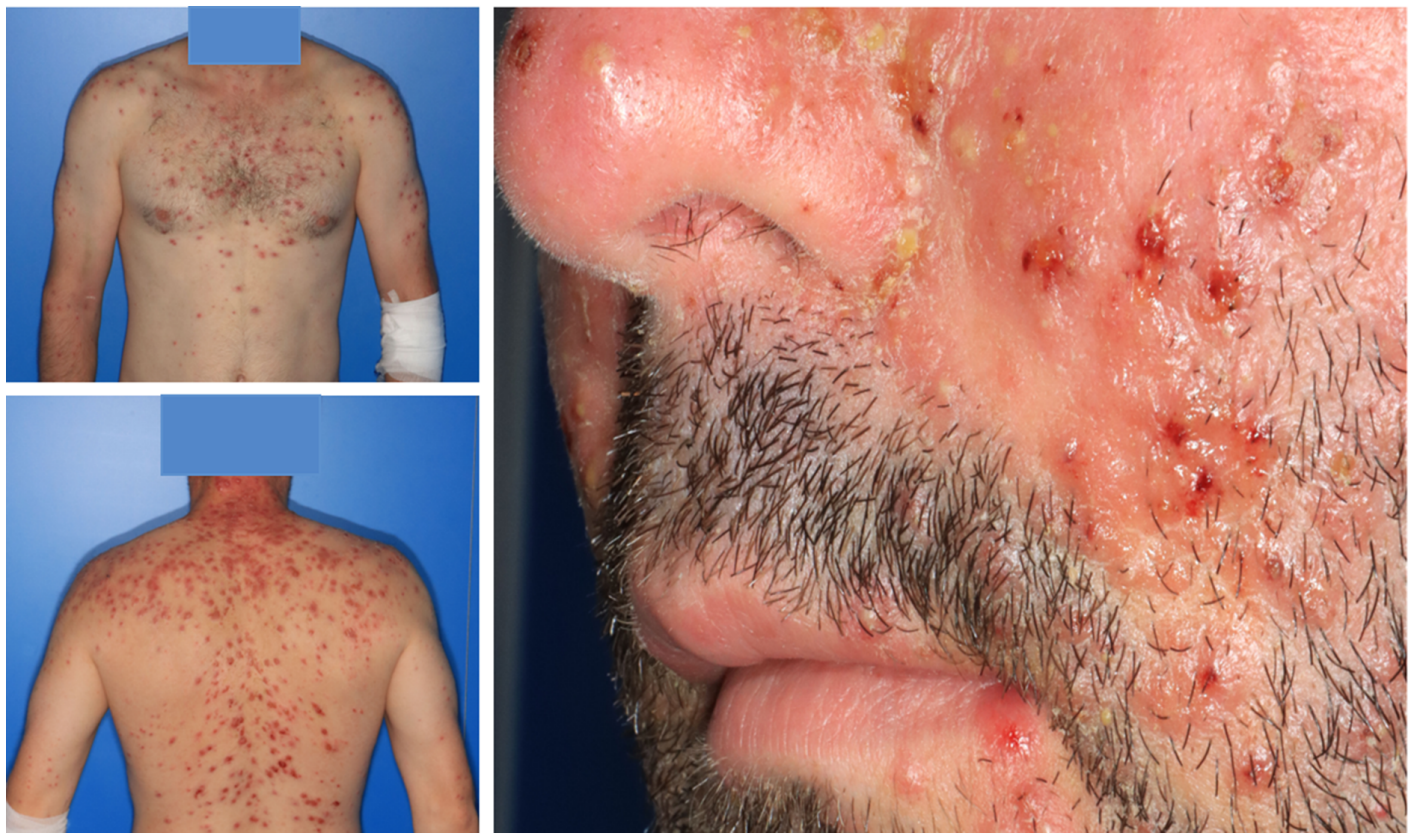
Infliximab-induced acneiform eruption with follicular papulopustular and nodulocystic lesions in a patient with Crohn’s disease, occurring 2 months after treatment initiation. Note involvement of the upper extremities and relative sparing of the forehead, which is unusual for acne vulgaris.

**Figure 2:**
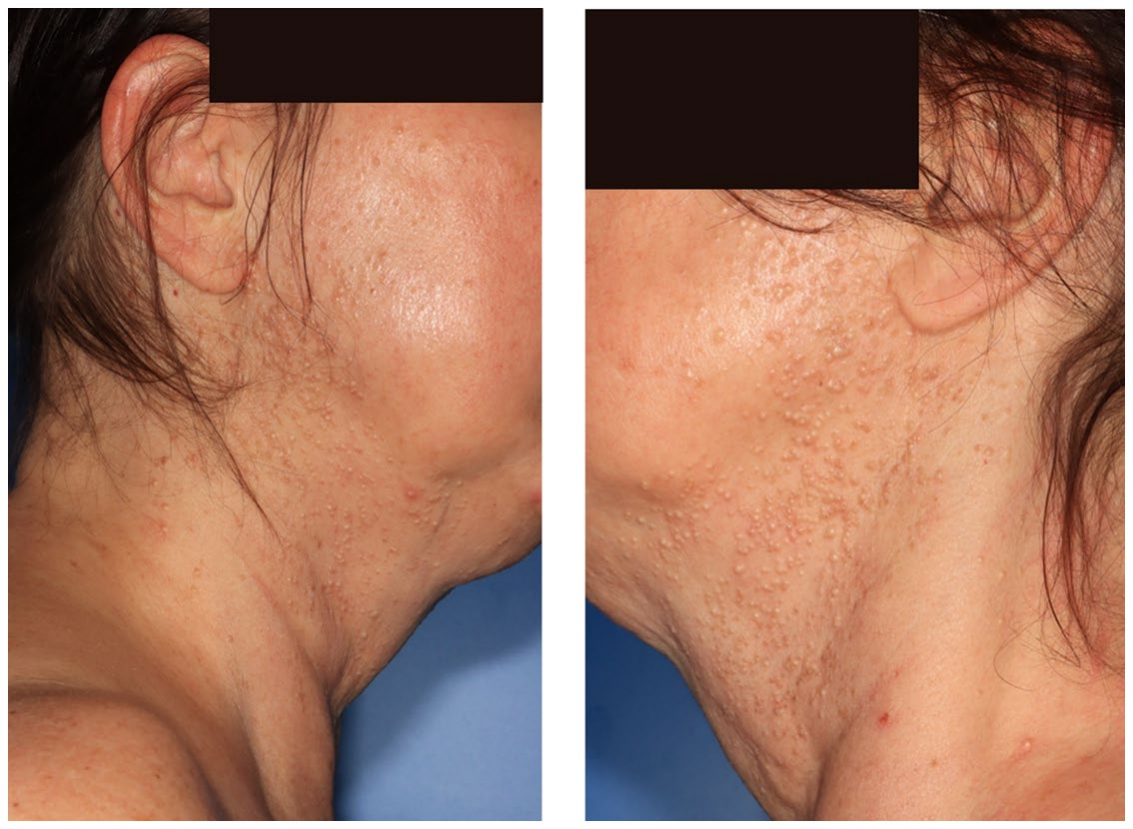
Imatinib-induced acneiform cADR with a monomorphic eruption of comedones and facial hyperplasia of sebaceous glands in a patient with *KIT*-mutated gastrointestinal stromal tumor (GIST), occurring 2 months after treatment initiation.

Although not life-threatening, acneiform ADRs tend to have a severe negative impact on the patient’s emotional and social activities, thereby impacting drug-adherence (3). There is no clear-cut definition of acneiform cADR; de-novo appearance of inflammatory lesions including follicular papules and pustules, sometimes comedones, involving areas rich in sebaceous glands (face, neck, upper back, chest, proximal extremities) with a temporal relation to a specific drug is a typical scenario (4) (Figures 1, 2). These follicular eruptions are estimated to comprise roughly 1% of all drug reactions with males being affected more often than females (1), rarely rendering drug interruption or discontinuation (3). Typical causing drugs include corticosteroids, androgens, anticonvulsants and anti-infective drugs as well as targeted antineoplastic drugs, best described for epidermal growth factor receptor (EGFR) inhibitors (5). Yet, a wide range of commonly prescribed drugs may rarely provoke this side effect as well, e.g., beta blockers such as propranolol (6). However, with a rapidly evolving landscape in drug-therapy, new triggers like januskinase (JAK) inhibitors are emerging as more common triggers for acneiform cADRs (7). Early recognition and appropriate treatment of acneiform eruption are important to enable clinicians to prevent more severe symptoms and discontinuation of therapy, thereby increasing patient compliance and optimizing clinical outcomes.

In this mini review, we aim to provide a comprehensive overview of clinical and histological findings of acneiform cADR as well as its distinction from other dermatoses, embedded in background information on pathophysiology as well as proposing a management algorithm based on available guidelines.

## 2 Characteristics of acneiform cADRs

### 2.1 Clinical findings

Acneiform cADRs typically develop weeks to months after initiating a new medication and, similar to acne vulgaris, predominantly appear in areas rich in sebaceous glands, including the face, upper trunk, and scalp. However, they may extend to non-seborrheic areas and involve atypical sites such as palms and soles. Notably, different agents can induce distinct clinical patterns of acneiform cADRs with nuanced differences (Table 1). Common symptoms include seborrhoea, burning sensations, pruritus, sometimes flushing and xerosis. Xerosis on the other hand may also be secondary in response to treatment approaches. Normally, there are neither laboratory abnormalities nor systemic involvement. EGFR-inhibitors may actually induce a range of symptoms involving skin, mucosa and adnexa apart from folliculotropic papulopustules, particularly paronychia, regulatory abnormalities of hair growth, itching and dryness, which is summarized under the acronym PRIDE (8).

**Table 1:** The frequency of acneiform cutaneous adverse drug reactions varies across drug classes, with the agents listed below representing the most common causative agents.

Drug class	Time of onset	Distribution	Morphological characteristics	Other symptoms/peculiarities
Glucocorticosteroids (including topical/oral/intravenous application)	2 weeks–several months	Seborrheic areas; extension to upper extremities possible	Monomorphic small, flesh-coloured or pink-to-red, dome-shaped inflammatory papules and pustules	Skin atrophy with extended duration of use; facial use of topical glucocorticoids may provoke perioral dermatitis
Androgens, anabolic steroids (e.g., testosterone, stanozolol, nandrolone, oxandrolone)	1 week–2 months	Seborrheic areas	Papulopustular and nodulocystic lesions; scarring possible	Gynecomastia, striae distensae, increased body mass index, decrease in testicular volume
Tuberculostatics/anti-TB-drugs (e.g., isoniazid, rifampicin)	5 weeks–18 months	Seborrheic areas	Sudden, extensive onset of open and closed comedones; inflammatory papules and pustules	More often in non-acne-prone adults or individuals without history of acne vulgaris
Halogens (e.g., iodides, bromides)	6–12 weeks	Lesions may occur everywhere; not restricted to seborrheic areas	Papulopustular and nodulocystic lesions; comedones	Occasionally mimicking pyoderma gangraenosum or ecthyma, hyperpigmentation, photosensitivity; chloracne: nose is spared by the eruption: “island in a sea aspect”
Immunosuppressants (e.g., cyclosporine, sirolimus)	2–4 weeks	Seborrheic areas; lesions can extend to the forearms, cervical area, and scalp	Papulopustular and nodulocystic lesions	Cyclosporine: hypertrichosis, epidermal cysts, keratosis pilaris, Sirolimus: predominantly males affected, especially those with a history of severe acne vulgaris.
TNF-alpha-inhibitors (e.g., infliximab, adalimumab)	1–4 months	Lesions may occur everywhere; not restricted to seborrheic areas	Papulopustular and nodulocystic lesions; sometimes comedones	
JAK-inhibitors (e.g., upadacitinib, abrocitinib, baricitinib, filgotinib, tofacitinib)	3–16 weeks	Seborrheic areas; especially face with a predilection to cheeks	Papulopustular and nodulocystic lesions; sometimes comedones	More frequent with JAK1-selective drugs, female predominance
TYK2-inhibitors (e.g., deucravacitinib, zasocitinib, envudeucitinib)	1–3 weeks	Seborrheic areas; especially head and neck; centro-facial distribution affecting the forehead, nose, perioral region, and chin	Papulopustular lesions; sometimes comedones	Female predominance
Bruton’s Tyrosine Kinase inhibitors (e.g., ibrutinib, zanubrutininb)	2 weeks–18 months	Seborrheic areas; especially face	Papulopustular lesions	Xerosis, petechiae, ecchymoses, photosensitivity, panniculitis, hair and nail changes
EGFR-inhibitors (e.g., cetuximab, erlotinib, gefitinib, osimertinib, panitumumab)	1–4 weeks	Seborrheic areas; may extend to extremities, palms, and soles	Papulopustular lesions; no comedones or cysts	Acneiform eruption is associated with favorable therapeutic outcomes (overall response rate, disease control); may occur with paronychia, regulatory abnormalities of hair growth, itching and dryness (PRIDE syndrome).
Her2-inhibitors (e.g., trastuzumab)	1–3 weeks	Seborrheic areas	Papulopustular lesions	Pruritus, xerosis, fissures, paronychia
VEGFR inhibitors (e.g., bevacizumab)	Within 2 weeks	Seborrheic areas	Erythematous papules; epidermoid cysts	Xerosis, hand–foot syndrome
BRAF-inhibitors (e.g., vemurafenib, dabrafenib, encorafenib)	1–3 weeks	Often non-seborrheic areas like extremities; seborrheic areas	Comedone-like lesions; papulopustular and nodulocystic lesions; rarely hidradenitis suppurativa –like lesions	Xerosis, photosensitivity, keratosis pilaris
MEK-inhibitors (cobimetinib, trametinib, binimetinib)	1–3 weeks	Seborrheic areas	Comedone-like lesions; papulopustular lesions; crusting	Xerosis, paronychia, cheilitis, photosensitivity, alopecia
PD-1-/PDL1-inhibitors (e.g., pembrolizumab, nivolumab, cemiplimab)	3–38 weeks	Trunk, extremities	Erythematous macules with or without papules	Pruritus, xerosis, vitiligo, rash
CTLA-4 inhibitors (e.g., ipilimumab, tremelimumab)	~3 weeks	Trunk, extremities	Papular erythematous rash	Pruritus, xerosis, vitiligo, rash

Notably, misuse of anabolic steroids or exposure to noxious agents may also cause acneiform eruptions (4, 5, 40, 43, 55–61). TNFi: tumor necrosis factor alpha inhibitors; JAK inhibitors: Janus kinase inhibitors; TYK: Tyrosinkinase Inhibitor; EGFR inhibitors: epidermal growth factor receptor inhibitors; HER2 inhibitors: human epidermal growth factor receptor 2 inhibitors; VEGFR inhibitors: vascular endothelial growth factor receptor inhibitors; BRAF inhibitors: B-Raf proto-oncogene serine/threonine kinase inhibitors; MEK inhibitors: mitogen-activated protein kinase kinase inhibitors; Multikinase inhibitors: multiple tyrosine kinase inhibitors; mTOR inhibitors: mammalian target of rapamycin inhibitors; PD-1 inhibitors: programmed cell death protein-1 inhibitors; CTLA-4 inhibitors: cytotoxic T-lymphocyte–associated protein 4 inhibitors.

It is very important to distinguish acneiform cADRs from more severe cutaneous drug reactions (SCARs). With an estimated incidence of 1 to 5 cases per million people per year, acute generalized exanthematous pustulosis (AGEP) is a rarer event. It is characterized by the abrupt onset of erythema with sterile, non-follicular pustules, typically appearing within 24–72 h after starting a new medication (90% of the cases), mostly beta-lactam antibiotics, and resolving by pin-point desquamation within 8 days once the agent is discontinued. Systemic symptoms like fever, neutrophilia, and eosinophilia are frequent (9). AGEP usually appears on the trunk and intertriginous regions (armpits, neck and groin), unlike acneiform reactions that characteristically affect areas rich in sebaceous glands. Notably, a localized variant (ALEP) usually involves the face and may be mistaken for acne by non-dermatologists.

Acne vulgaris is the most important differential diagnosis to acneiform ADRs. It typically presents with polymorphic lesions including comedones, papules, pustules, and nodules, with a predilection for seborrheic areas originally coining the term “acneiform distribution.” Acne vulgaris is one of the most common skin diseases worldwide. The highest prevalence is seen in adolescence with up to 85% of people between 13 and 25 years of age affected. Yet, it can persist or develop de-novo in adulthood as acne tarda, most often at ≥25 years of age. Flares of acne may occur in temporal relation to a new drug which may lead to confusion with cADRs and the best management. Standardized, questionnaire-based tools may help to determine the likelihood of drug-reaction, among others, the Naranjo Scale developed in 1981 or decision tree tools such as the Liverpool Adverse Drug Reaction Causality Assessment Tool (LCAT) (10, 11).

Another important differential diagnosis, especially in adulthood, is rosacea, which is characterized by persistent central facial erythema, telangiectasia, papules, and pustules, but lacks comedones. *Demodex* mite overgrowth is a central driver in the pathogenesis of rosacea, whereas in acne pathophysiology, *cutibacterium acnes* is of greatest importance, although demodex may contribute to the development of folliculitis (12). Diagnostic challenges arise in darker skin types due to the subtle nature of erythema; vascular and nociceptive symptoms may be more useful for making a definitive diagnosis of rosacea in these instances. However, as stated above, cADRs may also encompass burning or stinging sensations. Apart from that, perioral dermatitis needs mentioning, which presents as grouped erythematous papules and pustules around the mouth, sparing the vermillion border. It is more common in girls and young women. Comedones are usually absent. Acne excoriée des jeunes filles is defined by excoriated papules and postinflammatory changes due to habitual skin picking. It appears usually in young women, with evidence of manipulation and psychological distress (13). It should be borne in mind as differential diagnosis in atypical cases irresponsive to adequate treatment. Another pustular dermatosis potentially mimicking acneiform cADRs is eosinophilic folliculitis characterized by pruritic, grouped follicular papules and pustules, often on the face and upper trunk. It is more common in immunocompromised individuals and potentially associated to human immunodeficiency virus infection (14). Moreover, cutaneous lupus erythematosus (CLE) may rarely mimic acneiform eruptions, especially acute CLE with malar rash. Chronic discoid CLE typically presents with erythematous plaques, follicular plugging, and scarring, rarely papules may be folliculocentric. Cutaneous sarcoidosis presents with remarkable clinical heterogeneity as papules, nodules, or plaques, often with orange-yellowish areas and linear branching vessels on dermoscopy. It lacks follicular and comedonal features but may involve the central face (e.g., lupus pernio). The list of potential differential diagnoses is inexhaustive and we refer to common dermatology textbooks for other folliculocentric dermatoses. As drug history may be misleading, it is important to closely pay attention to clinical features to allow confident distinction. Still, histopathology will be needed in many instances.

### 2.2 Histology of acneiform cADR

Histologically, acneiform cADRs most commonly present as a superficial neutrophil-predominant suppurative folliculitis. The epidermis often shows spongiosis with occasional necrotic keratinocytes, while the dermis contains a perivascular and perifollicular mixed inflammatory infiltrate composed mainly of neutrophils and sometimes eosinophils, accompanied by mild papillary edema. The follicular apparatus typically reveals ectatic infundibula, focal rupture of the follicular epithelium, and dense neutrophilic infiltrates within and around the follicle (Supplementary Figure 1). Notably, comedones may be absent, helping to distinguish these reactions from classical acne vulgaris (15). In more severe cases, follicular rupture may lead to a granulomatous reaction with numerous histiocytes and multinuclear giant cells engulfing keratin material. AGEP as a relevant differential diagnosis is characterized by spongiform subcorneal and/or intraepidermal pustules filled with neutrophils. Key epidermal findings include necrotic keratinocytes, as well as spongiosis with neutrophilic exocytosis. In the dermis, prominent papillary edema is present together with mixed superficial, interstitial, and mid/deep-mixed infiltrates of neutrophils and eosinophils (16). Acne vulgaris, on the other hand, is defined histologically by more pronounced follicular hyperkeratinization, dilated follicles filled with keratin, sebum, and bacteria, and perifollicular lymphocytic and neutrophilic inflammation. Follicular rupture may lead to granulomatous inflammation and abscess formation. The presence of comedones remains a hallmark feature (17). Rosacea shows histopathologically no comedones, but follicular spongiosis and exocytosis of inflammatory cells, particularly lymphocytes, into hair follicles. The intensity of perifollicular inflammation and follicular inflammatory reactions are dependent on the severity of rosacea. The dermis reveals perivascular and perifollicular lymphohistiocytic infiltrates, sometimes accompanied by plasma cells. Vascular alterations such as telangiectasia and vascular proliferation are frequent, and *Demodex* mites may be present or abundant (18). Eosinophilic folliculitis is distinguished by a dense perifollicular and folliculotropic infiltrate of eosinophils and lymphocytes, with exocytosis of eosinophils into the follicular epithelium. The dermis often shows accompanying perivascular and periadnexal inflammation (14).

### 2.3 Pathophysiology of acneiform cADRs

Acneiform cADRs are best described in the context of EGFR- and Mitogen-activated protein kinase (MEK)-inhibitors. Satoh et al. (19) identified the skin commensal *Cutibacterium acnes* as a key driver of IL-36γ production in follicular keratinocytes. Transcriptomic analyses of affected skin revealed upregulation of IL-36γ, which occurs predominantly from keratinocytes within the hair follicle. IL-36γ is a proinflammatory cytokine known to drive expression of the neutrophil chemoattractant IL-8 (20). Given that both acne vulgaris and EGFRi-induced eruptions preferentially occur in areas with dense pilosebaceous units, a synergistic interaction between EGFR inhibition and *C. acnes*, which activates NF-κB through TLR2 signaling, is proposed. Combined exposure of primary human keratinocytes to *C. acnes* and EGFR inhibition resulted in a strongly enhanced induction of IL-36γ, an effect abolished by TLR2 siRNA. A similar treatment of human skin explants induced IL-8 in an IL-36γ–dependent manner and triggered neutrophil recruitment into hair follicles, resulting in folliculocentric pustular eruptions typical of acneiform cADRs (19). Satoh et al. further demonstrated that IL-36γ induction is mediated by binding of both NF-κB and the transcription factor Krüppel-like factor 4 (KLF4) to the IL36G promoter. KLF4 accumulates when the EGFR/MEK/ERK pathway is inhibited, as ERK1/2 normally promotes its polyubiquitination and proteasomal degradation. These mechanisms also explain the pustular inflammatory phenotype observed in individuals with genetic EGFR defects (19). Notably, drug-induced acneiform eruption is associated with favorable outcomes (better disease control and overall response rate) in cancer patients treated with EGFR-inhibitors (21). This was reported for lung cancer, colorectal cancer, pancreatic cancer and head and neck cancer. It resembles a robust pharmacodynamic marker of effective inhibition of the pathway. Effective on-target tumor activity correlates with on-target toxicitiy in the skin (5).

Alterations in sebum composition, triggered by corticosteroids, androgens, targeted therapies, and other drugs, lead to microbial imbalance and consecutive abnormal keratinocyte proliferation and differentiation. This contributes to follicular plugging and formation of inflammatory lesions. Overall increases in sebum production and shifts in lipid quality, such as enhanced squalene oxidation and changes in fatty acid profiles, create a lipid-rich and pro-inflammatory environment. This milieu promotes the overgrowth of pathogenic *C. acnes* phylotypes and reduces microbial diversity (22). The loss of *C. acnes* phylotype diversity and the overrepresentation of virulent IA1 strains directly contributes to Th17-mediated inflammation (23). IA1 strains are more efficient in stimulating early inflammatory responses, including the release of IL-1, IL-6, IL-8, and IL-17, thereby activating the Th17 pathway and driving neutrophil recruitment and pustule formation via IL17 and IL-22 (23) (Supplementary Figure 2).

Although the precise contribution of JAK-signaling to acne vulgaris remains uncertain, previous work has shown that JAK1 and JAK3 are overexpressed in acne lesions (24). JAK1-inhibitor-associated acneiform cADR seems to be dose-dependent and more severe in individuals with a prior history of acne vulgaris. A systematic review indicates that the incidence of drug-acne (“JAKne”) is highest with JAK1-selective agents, followed by JAK1/2 inhibitors, and lowest with TYK2 inhibitors (25). TYK2 inhibition, through attenuation of Th1 and Th17 pathways, may permit overproliferation of commensal microorganisms and subsequently induce inflammation of the pilosebaceous unit (26). Accordingly, selective TYK2 inhibition, e.g., by deucravacitinib, may divert inflammatory signaling toward alternative JAK pathways, particularly JAK1 and JAK3, thereby contributing to acneiform eruptions. Notably, both pan-JAK inhibitors and JAK1-selective inhibitors have been employed in the management of rosacea (27), raising the possibility that more than one mechanistic subtype of januskinase-associated acneiform eruption exists.

## 3 Management of acneiform ADR

When initiating medications with a known risk of acneiform cADRs, a structured risk assessment and the implementation of preventive skin care measures are essential. This should begin with a thorough personal and family history, including any previous or current inflammatory facial dermatosis such as acne vulgaris, rosacea, perioral dermatitis, or demodicosis. In addition, the abuse of anabolic–androgenic steroids and dietary habits should be reviewed, as high-glycaemic index foods may aggravate acne and related disorders. Baseline skin characteristics, including oily skin or seborrhoea, should be documented. Patients should also be questioned about their cosmetic routines, as greasy, occlusive, or comedogenic products, as well as over-the-counter topical corticosteroids, can increase the risk of acneiform eruptions and should therefore be avoided. For patients with acne vulgaris at baseline, dermatologic co-assessment is recommended when available. Should acneiform cADRs occur, several topical and systemic treatment options are available, as described in Table 2, allowing patients to continue their therapies in most cases. According to the Common Terminology Criteria for Adverse Events (CTCAE), pustular/acneiform ADR can be classified into five severity grades for which we provide management recommendations. Topicals can be used for mild to moderate grades (Grade 1 and 2). These include hydrophilic creams or gels containing comedolytic ingredients as well as daily gentle mechanical cleansing using syndets. Topical creams may include adapalene with or without benzoyl peroxide, azelaic acid, tretinoin, trifarotene, and combinations of benzoyl peroxide with clindamycin in cream or gel formulations. In Grade 2 reactions, oral antibiotics, like doxycycline or minocycline may be added. In severe cases (Grade 3 and 4), oral isotretinoin or systemic prednisone and dose adjustment or discontinuation of the causing agents should be considered. Red flags include mucosal involvement, blistering, erosions, fever, systemic symptoms and rapidly spreading erythema as well as difficulties in ruling out SCARs. In the setting of tuberculostatic treatment, typical triggers of acneiform cADRs include rifampicin and isoniazid; to allow sufficient duration of treatment, discontinuation of the offending agent and switch to an alternative may be discussed based on resistance situation. However, similar to patients receiving antineoplastic therapies, maintaining treatment may be crucial and feasible adhering to the above-mentioned principles.

**Table 2:** Treatment of pustular drug eruption, algorithm adapted from the common terminology criteria for adverse events (CTCAE) v5.0 and v6.0 (62–65).

Grade	Definition	Management
Grade 1	Papules and/or pustules covering <10% BSA, with or without pruritus or tenderness.	Topicals: Light hydrophilic creams or gels; comedolytic ingredients (retinol, lactic acid, fruit acids) Daily gentle mechanical cleansing; syndet instead of soap; micellar water if using makeup; optional mild chemical peels (glycolic/salicylic acid) Adapalene +/or benzoyl peroxide; azelaic acid Tretinoin; trifarotene; benzoyl peroxide and clindamycin cream/gel
Grade 2	Papules and/or pustules covering 10–30% BSA, with or without pruritus or tenderness; associated with psychosocial impact; limiting instrumental ADL or severe impact on age-appropriate normal daily activity	Same topicals as Grade 1. Oral doxycycline (40–100 mg per day) or minocycline (50–100 mg per day); Macrolides may be considered in specific situations (e.g., allergy); note that cytochrome-mediated interactions with JAK inhibitors are possible
Grade 3	Papules and/or pustules covering >30% BSA, with or without pruritus or tenderness; limiting self-care ADL or severe impact on age-appropriate normal daily activity; associated with local superinfection requiring oral antibiotics.	Same as Grade 2. Consider oral isotretinoin (0.1–0.5 mg/kg) or prednisone (0.5 mg/kg); not as effective as antibiotics in recent studies Reduce targeted agents or causing drug per label.
Grade 4	Papules and/or pustules covering any % BSA, with or without pruritus or tenderness; extensive superinfection requiring IV antibiotics. life-threatening consequences.	Same as Grade 3. Discontinue targeted agents or causing drug as soon as possible.
Grade 5	Death	–

BSA: body surface area; ADL: activities of daily living.

A meta-analysis demonstrated that prophylactic treatment with oral tetracyclines significantly reduces the incidence of anti-EGFR–induced acneiform adverse drug reactions. Patients receiving prophylactic antibiotics had an approximately 70% lower risk of moderate to severe reactions (grades 2–4). A significant reduction in skin reactions was observed only in studies using minocycline. Nevertheless, efficacy of minocycline and doxycycline is comparable in the treatment of acne vulgaris, and we prefer doxycycline in clinical practice because of its safety profile (28). In another study, 17 patients treated with modified-release, subantimicrobial-dose doxycycline (40 mg) while receiving EGFRi, MEKi or tyrosine kinase inhibitors were analysed. In 47% of patients, the ADR improved by at least one CTCAE grade, while 29% showed stabilization of symptoms and 24% showed no clinical benefit. The patients treated with EGFR-directed monoclonal antibodies (cetuximab, panitumumab) were non-responders and were switched to the full antimicrobial dose of doxycycline (200 mg / day) and responded subsequently. A secondary infection might explain the insufficient response to subantimicrobial doxycycline in these cases. The authors recommended switching to full-dose doxycycline after 28 days, if 40 mg doxycycline is ineffective (29).

## 4 Overview of drugs associated with acneiform cADR and mechanisms involved

Acneiform cADRs comprise a heterogeneous group of drug-related follicular eruptions that differ from acne vulgaris. Grouping these reactions by drug class and the underlying pathophysiological mechanism helps clinicians anticipate the clinical course and choose appropriate management strategies (Tables 1, 2).

### 4.1 Topical drugs

Oil-based or occlusive topicals are known to cause acne comedonica by promoting follicular hyperkeratinization and plugging. This process is independent of systemic drug exposure and might be thought of as an exogenous acneiform reaction rather than an actual cADR (30, 31). To retain brevity, we do not further elude on this topic, as it is out of scope of this mini-review.

### 4.2 Non-targeted systemic agents

Drugs such as corticosteroids, androgens, lithium, anticonvulsants and anti-infectives induce acneiform lesions through several mechanisms, including increased sebum production, changes in neutrophil migration or shifts in inflammatory cytokines like IL-8 or TNF-*α* (4, 30, 31). The timing of onset varies considerably, often appearing weeks or even months after therapy initiation. Lesions are usually monomorphic and may include comedones in lithium- or androgen-associated eruptions. The distribution is mainly seborrheic, though lithium and halogens can involve non-seborrheic sites as well. Most of these reactions respond to standard acne treatments, including tetracyclines, although clinical improvement sometimes requires reducing or discontinuing the trigger, particularly in corticosteroid- or lithium-associated cases (1).

### 4.3 Targeted systemic agents

Acneiform reactions to targeted systemic agents typically appear soon after treatment initiation, often within one to 3 weeks (19). They interfere with epidermal growth factor receptor signaling, disturb keratinocyte differentiation and weaken barrier and innate immune function, which facilitates IL-36γ- and IL-8-mediated recruitment of neutrophils (32, 33). The resulting papulopustular lesions are highly inflammatory, tender and characteristically lack comedones. They are most often located on the face, scalp and upper trunk, although some patients also develop palmoplantar involvement. Her2-inhibitors exert similar downstream inflammatory effects (34), and CFTR-modulators can lead to crystalline folliculitis (35). Immune checkpoint inhibitors seldom cause acneiform eruptions, and in those cases, nonspecific T-cell activation is assumed to play a role (36, 37).

### 4.4 Small molecule kinase inhibitors

Agents that inhibit intracellular signaling, including JAK-, TYK2-, Bruton’s tyrosine kinase (BTK-) and multikinase tyrosine kinase inhibitors (TKIs), can lead to acneiform inflammation through modulation of the JAK–STAT axis or off-target inhibition of kinases such as EGFR, SRC or ITK (38). The time to onset generally lies between two and 12 weeks, although BTK inhibitors may deviate from this pattern. The eruptions are monomorphic and neutrophil-rich and often appear on the face or scalp; TKIs can show a more variable distribution of lesions (39). JAK1-selective agents and TYK2 inhibitors have shown the highest incidence in clinical studies (40–42). Treatment usually follows protocols for acne vulgaris even though some authors consider JAK-induced acneiform ADRs to resemble rosacea. JAK- or TYK2-associated eruptions are often mild, whereas reactions to BTK inhibitors or certain TKIs may require more intensive therapy, including isotretinoin.

## 5 Discussion

Despite improved characterization of typical clinical patterns and histopathological hallmarks, several questions remain unanswered. The relevance of follicular keratinization, sebum composition and microbiome changes differs across drug classes and is not fully defined. JAK–STAT inhibition is a particular challenge, as these agents may improve severity of inflammatory dermatoses in some contexts but provoke acneiform reactions in others (43). Moreover, solid diagnostic criteria that differentiate acneiform cADRs from acne vulgaris and other inflammatory facial dermatoses aggravated in temporal relation to drugs are still to be defined. Taking a medication history is essential, especially to identify any newly started drugs in the last days up to months. Evidence from pediatric, geriatric, and skin-of-color populations remains limited. Misclassification continues to be frequent in both clinical trials and routine care, and heterogeneous outcome measures further hinder meaningful comparison across studies (1, 44–46).

Evidence based recommendations for management remain limited. Most approaches rely on expert consensus or acne vulgaris guidelines rather than dedicated clinical trials. The optimal dosing and duration of systemic therapy, the role of prophylaxis and the long-term impact on adherence to essential systemic treatments are not well established (1, 47). Existing studies often include only small cohorts and have short follow up periods.

Newer approaches using pharmacovigilance databases and machine learning may help identify drug specific risk patterns and improve stratification (48, 49). Advances in molecular dermatology are providing deeper insight into cytokine networks, neutrophil trafficking and microbiome interactions, and may support more precise diagnostic and monitoring tools in the future (50, 51). Ultimately, treatment strategies are likely to move toward individualized, mechanism-oriented approaches that maintain essential systemic therapies whenever possible, supported by preemptive skin care and close collaboration between dermatology and the prescribing specialties (52–54).

In this review, we provide an overview of the most common causes of acneiform ADRs and show recommendations for appropriate therapeutic management. It is worth considering whether, when initiating drugs commonly associated with acneiform ADRs, the patients should be treated proactively with topical or even systemic acne treatments to prevent severe flares and potentially avoid treatment discontinuation. Until today, this decision will be made on a case-to-case basis.

## Data Availability

The data that support the findings of this study are available upon reasonable request from the corresponding author.
