# Supplementary figures and images for: Acneiform drug eruptions—update on pathophysiology and culprit drugs
Source: Front Med (Lausanne). 2026 Feb 23;13:1769362. doi: 10.3389/fmed.2026.1769362 (PMC12969058; doi:10.3389/fmed.2026.1769362)

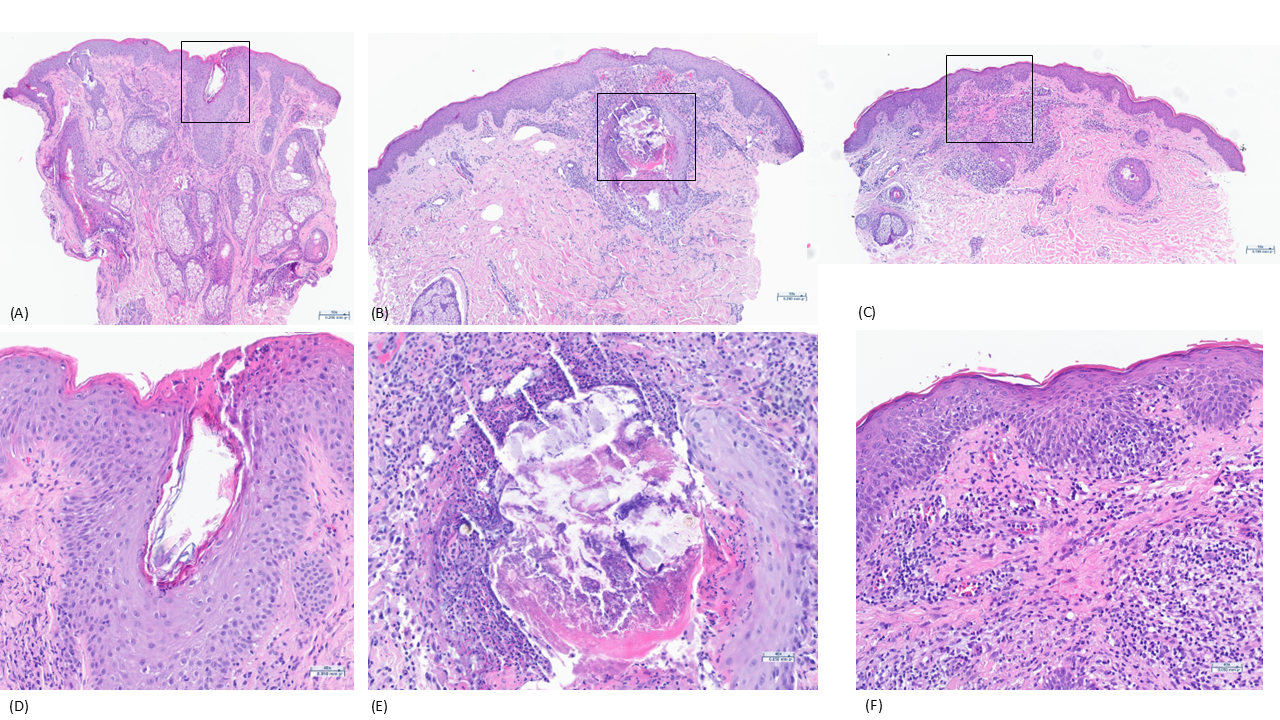

Supplement: Supplementary file 1 [file Image_1.tif]

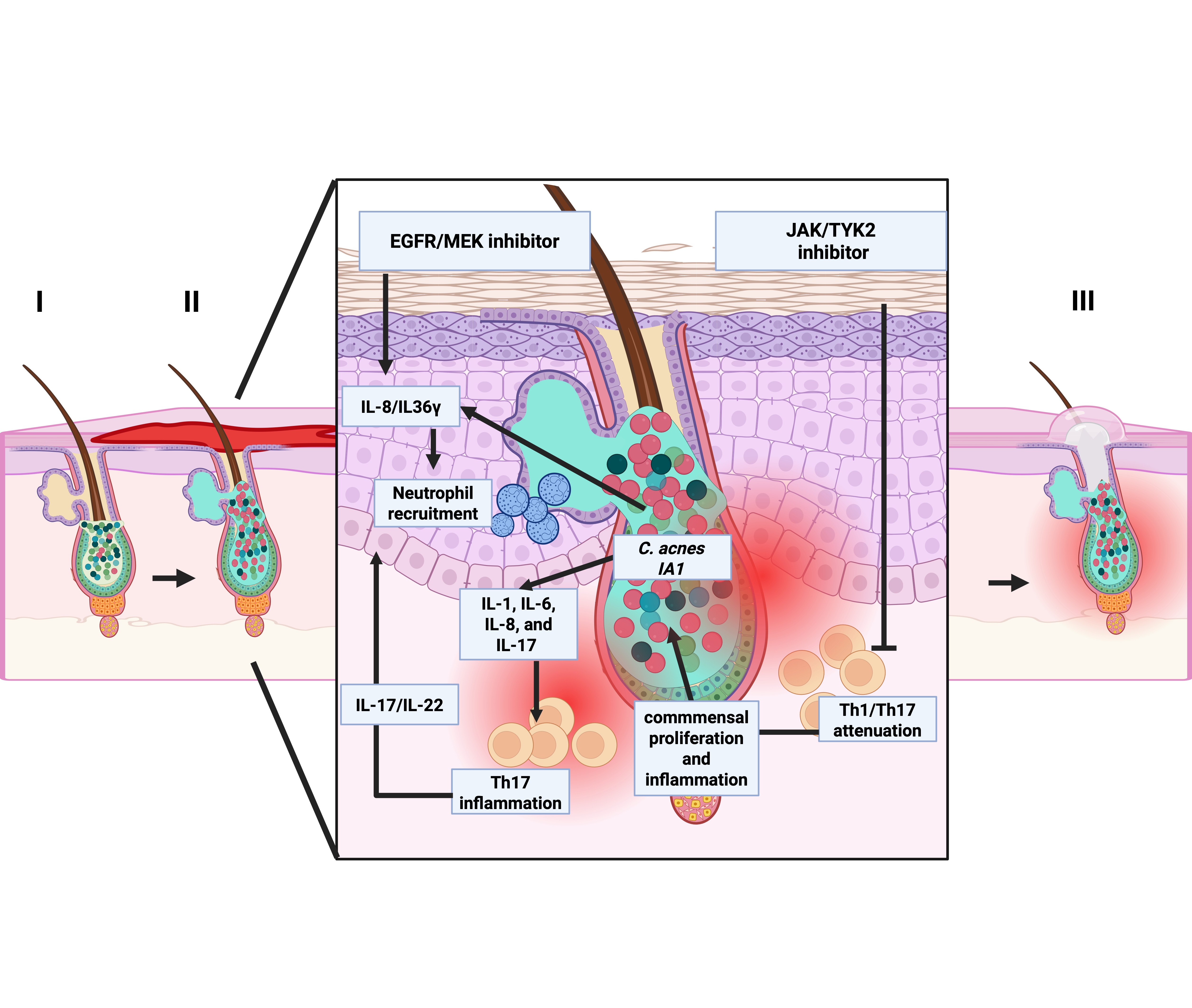

Supplement: Supplementary file 2 [file Image_2.jpeg]
