# Supplementary material for: Acneiform drug eruptions—update on pathophysiology and culprit drugs
Source: Front Med (Lausanne). 2026 Feb 23;13:1769362. doi: 10.3389/fmed.2026.1769362 (PMC12969058; doi:10.3389/fmed.2026.1769362)
Supplement: Supplementary file 3 [file Supplementary_file_1.docx]

**Supplementary Figure 1:** Histopathological findings of acneiform eruptions are variable and often non-specific. They may comprise a mixed neutrophilic and lymphocytic infiltrate with folliculitis / perifolliculitis, epidermal changes such as parakeratosis, acanthosis and spongiosis as well as admixed eosinophils. Severe cases may lead to follicular rupture with subsequent histiocytic foreign body reaction to keratin. A+D) Male, 68 years, forehead; B+E) Male, 42 years, upper back; C+F) Female, 71 years, upper back. A-C: H&E stain, 100x original magnification; D-F: H&E stain, 400x original magnification.

**Supplementary Figure 2:** Pathophysiology of acneiform cutaneous adverse drugs reactions. I: Normal follicular apparatus with commensal microorganisms, including *Cutibacterium acnes* *(C. acnes)*; II: Various non-targeted or targeted therapies (e.g. EGFR-, MEK-, JAK- or TYK2-inhibitors) may change sebum composition, promote follicular hyperkeratinization resulting in follicular plugging, and overproliferation of pathogenic *C.acnes*phylotypes. These changes promote neutrophil recruitment, and a Th17-dominant milieu. III: Subsequent changes may include formation of folliculotropic pustules resulting in the typical clinical appearance. Created in BioRender. Maisch, T. (2026) <https://BioRender.com/576e9vl>
